# A Review of the Surface Modification of Cellulose and Nanocellulose Using Aliphatic and Aromatic Mono- and Di-Isocyanates

**DOI:** 10.3390/molecules24152782

**Published:** 2019-07-31

**Authors:** Hatem Abushammala, Jia Mao

**Affiliations:** Fraunhofer Institute for Wood Research (WKI), Bienroder Weg 54E, 38108 Braunschweig, Germany

**Keywords:** cellulose, nanocellulose, isocyanate, surface, modification, functionalization

## Abstract

Nanocellulose has been subjected to a wide range of chemical modifications towards increasing its potential in certain fields of interest. These modifications either modulated the chemistry of the nanocellulose itself or introduced certain functional groups onto its surface, which varied from simple molecules to polymers. Among many, aliphatic and aromatic mono- and di-isocyanates are a group of chemicals that have been used for a century to modify cellulose. Despite only being used recently with nanocellulose, they have shown great potential as surface modifiers and chemical linkers to graft certain functional chemicals and polymers onto the nanocellulose surface. This review discusses the modification of cellulose and nanocellulose using isocyanates including phenyl isocyanate (PI), octadecyl isocyanate (OI), toluene diisocyanate (TDI), diphenylmethane diisocyanate (MDI), hexamethylene diisocyanate (HMDI), and their derivatives and polymers. It also presents the most commonly used nanocellulose modification strategies including their advantages and disadvantages. It finally discusses the challenges of using isocyanates, in general, for nanocellulose modification.

## 1. Introduction

**Figure 1:**
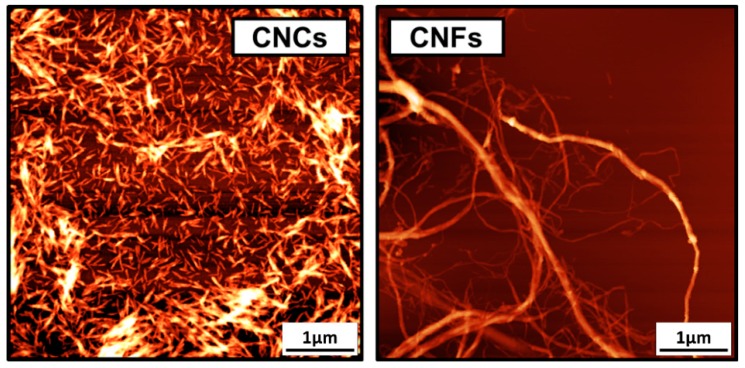
Atomic Force Microscopy images (5 × 5 µm) of CNCs and CNFs. (The images were obtained by the authors using Agilent 5500 AFM (Keysight Technologies, Santa Rosa, CA, USA) for CNC and CNF samples purchased from the University of Maine).

**Figure 2:**
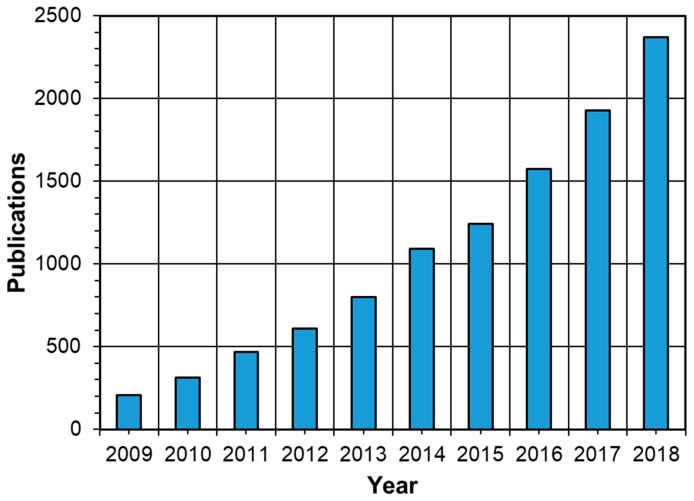
The number of publications on nanocellulose in the last decade indicating the increasing interest in nanocellulosic materials (Web of Science, July 2019, nanocellulose; cellulose nanocrystals/whiskers/fibers/fibrils; nanocrystalline cellulose; micro/nanofibrillated cellulose).

Cellulose is Earth’s most abundant biopolymer. It represents 40–60% of wood mass and can be extracted in the form of 20–40 µm thick fibers upon pulping [1,2]. Apart from its traditional use in the paper and packaging industries and its growing conversion into textile fibers, cellulose can be processed into functional nanoparticles [3]. Among these, cellulose nanocrystals (CNCs) are crystalline nano-rods with a thickness of 3–10 nm and a length of few hundred nanometers (Figure 1) [4]. They are extracted from pulp fibers using an acid-mediated procedure, which has already been industrialized [5]. They can also be extracted directly from wood and lignocelluloses using a variety of reagents and processes [6,7,8]. CNCs with a length in the micrometers can also be obtained from tunicate cellulose [9]. Cellulose nanofibrils (CNFs), another form of nanocellulose, are semi-crystalline spaghetti-like nanoparticles with a thickness of 5–30 nm and a length of few micrometers (Figure 1) [4]. They are produced by the mechanical fibrillation of pulp fibers using a wide range of techniques including microfluidization and homogenization [3]. In addition to their high mechanical properties, biodegradability, and high surface area, CNCs and CNFs are famous for the possibility to modify their surfaces through the abundant hydroxyl groups. They can also orient themselves in 1D, 2D, and 3D nanostructures forming functional liquid crystalline structures [10]. Due to these interesting properties, CNCs and CNFs have shown great potential in a wide range of applications including automotive industry [11], drug delivery [12], tissue engineering [13], packaging [14], and water filtration [15]. As a result, the number of publications and patents on nanocellulose per year increased from 208 in 2009 to 2372 in 2018 (Figure 2). This has also led to the establishment of more than 20 nanocellulose production facilities in the last two decades such as CelluForce, Innventia, and Blue Goose Biorefinaries [3].

The surface modification of nanocellulose through its hydroxyl groups has significantly increased its potential. A wide range of chemical functionalities has been placed on nanocellulose surface through simple reactions such as oxidation and acetylation [16,17]. Sometimes, the reaction involved the grafting of functional materials and polymers onto the surface. These modifications aimed at modulating the surface properties of nanocellulose to improve its processing with nonpolar matrices [18,19,20], or to change its affinity to certain polar and nonpolar molecules [21,22]. Other times, the modification aimed at placing functional groups onto the nanocellulose surface to target certain applications [23]. In this case, a chemical linker was often needed to bind these functional groups to nanocellulose, whether they were simple compounds or polymers. Among many modifiers and linkers, aliphatic and aromatic isocyanates have attracted an increasing attention in the recent years for nanocellulose modification although they have been used for a century with cellulose. They are a very interesting group of chemicals that are most famous for making polyurethanes through their reactions with polyols [24].

**Figure 3:**
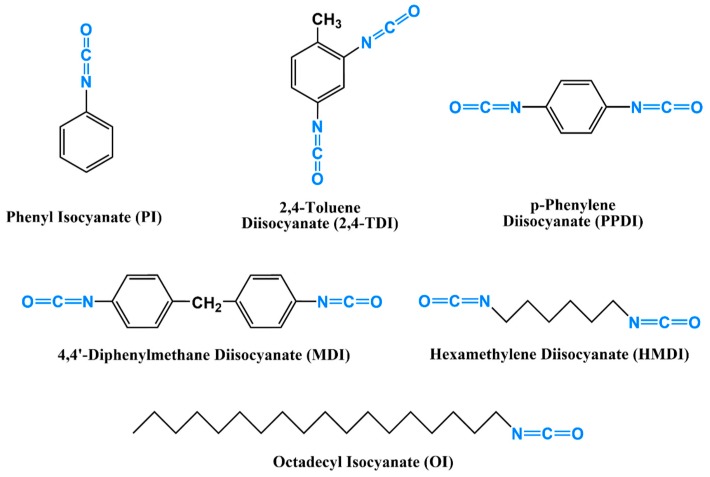
The most commonly used aromatic and aliphatic mono- and di-isocyanates for cellulose and nanocellulose modification.

Aliphatic and aromatic mono-isocyanates such as phenyl isocyanate (PI) and n-octadecyl isocyanate (OI), and di-isocyanates such as toluene diisocyanate (TDI), diphenylmethane diisocyanate (MDI), hexamethylene diisocyanate (HMDI), and their polymeric forms have all been used for the modification of cellulose and nanocellulose (Figure 3) [25,26,27,28]. PI and OI were mainly used to decrease the hydrophilicity of cellulose and nanocellulose as they do not have the necessary second isocyanate to function as a linker, while TDI, PPDI, MDI, and HMDI have been used both as surface modifiers and chemical linkers [29,30]. TDI, MDI, and HMDI differ mainly in their molecular rigidity, which is mainly dependent on the benzene rings. MDI is the most rigid because of the two benzene rings while HMDI is the most flexible because of the aliphatic chain. This, for sure, has a significant impact on the mechanical properties of the materials of which they become a part [31]. For instance, the MDI-based polyurethane foams are more rigid than those made using TDI [32].

TDI is a very interesting di-isocyanate as the isocyanates of its 2,4-isomer differ in their reactivity. The ortho isocyanate is 5–10 time less reactive than the para isocyanate due to the steric hindrance from the neighboring methyl group [33,34]. This makes 2,4-TDI very advantageous for binding materials to each other as one material can selectively react with the para isocyanate followed by the reaction of the other material with the ortho one. In addition to price, this explains the less frequent use of 2,6-TDI. TDI is often sold as an 80%/20% (2,4/2,6) mixture of its isomers. 

To facilitate the reaction between isocyanates and the hydroxyl groups of nanocellulose and the resultant formation of polyurethane bonds, amines such as triethylamine were found to be excellent catalysts although they could also facilitate the self-polymerization of isocyanates as a side reaction [35].

This review discusses the use of aliphatic and aromatic mono- and di-isocyanates in their molecular and polymeric forms for the surface modification of both cellulose and nanocellulose. For each material, the literature reports will be categorized based on the goal of the surface modification. Some of these reports followed application-oriented approaches, which focused on modifying cellulose and nanocellulose to function in certain applications while other reports were property-oriented, which aimed at improving the properties of cellulose and nanocellulose in general, such as hydrophobicity and thermal stability, without targeting a specific application. Most of the reports; however, focused on the use of isocyanates to facilitate the processing of cellulose and nanocellulose in nonpolar thermoplastic and thermoset matrices to prepare reinforced composites with improved interfacial adhesion.

## 2. General Scenarios of Nanocellulose Modification Using Isocyanates

**Figure 4:**
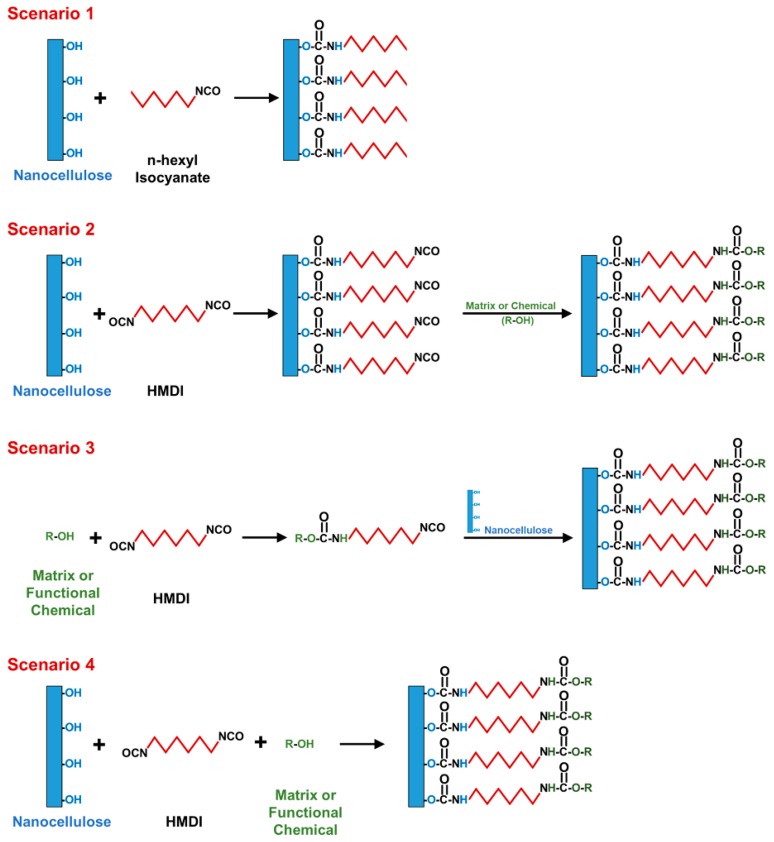
Overview of the use of isocyanates and di-isocyanates for nanocellulose modification (hexyl isocyanate and HMDI as examples).

Most of the reported reactions between nanocellulose and isocyanates took place through one of four scenarios. The first scenario represents the reaction of nanocellulose with a mono-isocyanate carrying either a hydrophobic (alkyl or aryl) [36,37] or a functional group [38] (Figure 4). In case of hydrophobization, the modified nanocellulose is usually blended/mixed with a nonpolar matrix to produce a nanocomposite [39,40]. The matrices in this scenario were mainly thermoplastic petroleum-based polymers such as polystyrene, polypropylene, and polyethylene. Sometimes, the reaction between the isocyanate and nanocellulose took place while processing both with the thermoplastic polymer [41]. Scenario 1 was rarely used with di-isocyanates as there is no need for a second isocyanate group in this scenario [42]. Some of those rare cases assumed that both isocyanates reacted with the nanocellulose (cross-linking). 

Di-isocyanates were mainly used to (1) bind functional groups onto the nanocellulose surface or to (2) support its processing with thermoset matrices. In the first case, two scenarios were used (Figure 4). In Scenario 2, the di-isocyanate is reacted with nanocellulose through one of its isocyanates and the free isocyanate is reacted then with a functional polymer or molecule [43]. Sometimes the free isocyanate is reacted with a non-functional alcohol to obtain a hydrophobic nanocellulose [44]. In Scenario 3, the di-isocyanate is reacted with the functional material or polymer at first then the free isocyanate is reacted with the nanocellulose [45]. This scenario seems to be more efficient than Scenario 2 in general as some reports have shown that di-isocyanates, following Scenario 2, could react with nanocellulose using both of their isocyanates making them dysfunctional for the second stage of the reaction [46,47]. Scenario 4 is the most commonly used for the processing of nanocellulose with thermosets such as polyurethanes (Figure 4). In this scenario, a mixture of the nanocellulose, di-isocyanate, and the polyol are cross-linked together at the same time [48,49]. It is important to mention that these fours scenarios have been used similarly for both cellulose and nanocellulose.

## 3. Cellulose Modification Using Aliphatic and Aromatic Isocyanates

The first report on the use of isocyanates with cellulose seems to go back to 1920 by Charles [50] followed by other reports focusing on the use of the reaction for textile industry to produce cellulose fibers with improved mechanical and thermal properties [51,52,53]. In 1962 and 1970 the reaction between cellulose and isocyanates was further explored by Ellzey et al. and Ohno et al. respectively, with a focus on PI [54,55]. Ohno later studied the reaction of cellulose with di-isocyanates (2,4-TDI and HMDI) [56,57]. Isocyanates have then become more frequently used to modify cellulose for different purposes (Table 1), which can be categorized into:

### 3.1. Preparation of Functional Cellulose (Scenario 1 and 2)

Cellulose and its derivatives have been functionalized for a variety of applications including water treatment, chromatography, and biotechnological applications. In terms of the biotechnological applications, Gemeiner et al. used in 1977 the reaction between cellulose and 2,4-TDI to prepare cellulose isothiocyanates, whose binding capacity to thiol and amine compounds was studied [58]. The aim was to explore its potential for enzyme immobilization and for solid-phase sequence analysis of peptides and proteins. In the same year, cellulose beads, upon their reaction with 2,4-TDI or HMDI, were also explored for enzyme immobilization [59]. Dierov et al. developed a hydrophobic cellulose-based sorbent to collect the lipase secreted from a fungus (rhizopus–microporous), by reacting microcrystalline cellulose (MCC) with 2,4-TDI through the para isocyanate followed by reacting the ortho isocyanate with n-butanol [44]. A cellulose-based sorbent was also prepared using cyclohexyl isocyanate or PI to be used for the extraction of natural estrogenic hormones [60]. Pend et al. functionalized cellulose using a phosphonium-containing isocyanate to obtain a product which had both antibacterial and antifungal activities against certain microorganisms [61]. Similarly speaking, the reaction of sulfopropylbetaine or quaternary ammonium salt carrying a reactive isocyanate with cotton cellulose led to a cotton imparting excellent antifouling and bactericidal activity with enhanced hydrophilicity, biocompatibility, and mechanical properties [38].

There are two reports on cellulose functionalization for water filtration. In 1983 Sato et al. used the reaction between cellulose and 2,4-TDI to graft amino acids, such as glycine and serine, onto the cellulose to produce a powder capable of adsorbing heavy metals such as copper, zinc, and cadmium [62,63]. Recently in 2018, cellulose was reacted with MDI to develop a sorbent for the remediation of hydrocarbon-polluted water [64].

To developed chiral stationary phases for liquid chromatography, cotton spheres were reacted with HMDI, 2,4-TDI, 1,4-MDI, or substituted PIs [26,27,28]. Chiroptical cellulose was also obtained upon its reaction with 3-chlorophenyl isocyanate or 4-chlorophenyl isocyanate, which formed with silica chiral nematic mesomorphic structures [65].

### 3.2. Improving Cellulose Properties (Scenario 1 and 2)

In this category, cellulose was treated with isocyanates to mainly reduce its hydrophilicity or improve its biocompatibility. To reduce it hydrophilicity, cellulose was reacted with PI, cyclohexyl isocyanate, HMDI, and oxime-blocked isocyanate oligomers [25,66,67]. For the same goal, Botaro et al. followed a more complicated approach, by which the cellulose fibers were reacted with alkenyl isocyanate followed by its radical polymerization with other monomers (styrene or methylacrylate) to grow hydrophobic polymeric brushes around the fibers [68,69]. A more-direct approach by Trejo-O’Reilly et al. reacted cellulose with an isocyanate-containing polystyrene [70,71]. Badanova et al. hydrophobized a cellulose fabric by immersing it in an aqueous solution of polyethylene glycol followed by impregnation in an organic solution of 2,4-TDI and thermal pressing [72]. 

Isocyanates were also used to improve cellulose biocompatibility with human blood. For instance, cellulose sheets were reacted with HMDI through one of its isocyanates followed by the reaction of the other isocyanate with betaine-containing molecules [73]. Similarly, a cellulose fabric was reacted with 2-methacryloyloxyethyl isocyanate to allow a following grafting of phosphoryl choline, which improved the biocompatibility of the fabric to function as a hemodialysis material for blood purification [74]. To improve their mechanical, chemical, and thermal properties, cellulose acetate membranes were reacted with phenyl, propyl and butyl isocyanates [75,76].

### 3.3. Improving Cellulose Processing and Performance with Nonpolar Matrices (Scenario 1 and 3)

In this category, cellulose was either reacted with the isocyanate prior to mixing/compounding with the nonpolar matrix or the reaction took place during compounding by relying on the heat used in the process. Mono-isocyanates, di-isocyanates, and their polymers have all been used in this category while the matrices were mainly petroleum-based thermoplastic polymers such as polystyrene (PS), polypropylene (PP), and polyethylene (PE). The modification led in most of these reports to a significant improvement in the mechanical properties and a drop in the water uptake of the composite. 

Cellulose was modified using poly(methylene(polyphenyl isocyanate)) and MDI to improve the interfacial adhesion between cellulose and PS [77,78,79] while the interfacial adhesion in cellulose/PP composites was improved by reacting cellulose with different alkyl isocyanates [80], 2,4-TDI [81], and HMDI [82]. A PP/PLA composite was reinforced by birch pulp fibers after modifying the fibers with 2,4-TDI. The modification improved the mechanical and thermal properties, water resistance of the composite, and its stability upon weathering [83]. In the case of cellulose/PE composites, cellulose was modified using poly(methylene(polyphenyl isocyanate)), HMDI, and OI before compounding [69,84,85,86]. For the same purpose, sisal fibers were functionalized following Scenario 3 using cardanol and 2,4-TDI before compounding with PE [87,88]. In some cases, the isocyanate is used directly as a compatibilizer. For instance, polybutadiene isocyanate and derivatives of MDI were used as compatibilizers in cellulose/PP composites [41,89]. It is assumed that the isocyanate and cellulose reacted during the processing of the composite.

Cellulose has also been modified before being used to reinforce bio-based matrices such as natural rubber [90], epoxidized soybean oil polymer [91], polyesters [92], thermoplastic polyurethanes and polyamides [93,94], and castor oil [95,96]. In few reports, cellulose was modified using OI to improve its processing and stability in fiber-reinforced cement [97,98]. 

### 3.4. Cellulose/Matrix Cross-linking (Scenario 3 and 4)

The reports in this category used di-isocyanates (mainly 2,4-TDI) for cross-linking cellulose with the matrix. In some cases, the matrix was reacted with one of the isocyanates of the di-isocyanate then the cellulose was reacted with the free isocyanate (Scenario 3). For instance, poly(caprolactone) (PCL) was reacted with 2,4-TDI then cellulose was reacted with the free isocyanate to form the composite [99]. Copolymers of cellulose acetate with poly(caprolactone monoacrylate) or poly(butylene glycol adipate) were prepared using the same procedure [100,101]. In other cases, cellulose was reacted with the di-isocyanate then cured together with the matrix, which was made of the same di-isocyanate and a polyol such as lignin or castor oil. For instance, MCC was treated with MDI then added to castor oil and MDI to cure all together for forming the composite [102]. Sometimes the cellulose was added directly without modification to the polyol/di-isocyanate mixture then cured together (Scenario 4) [103]. Clearly, cellulose modification prior to curing improved the mechanical performance of the composite more significantly than without it.

## 4. Nanocellulose Modification Using Aliphatic and Aromatic Isocyanates

Despite their use for cellulose modification for almost a century, isocyanates were used for the first time in 2008 to modify nanocellulose [104]. Two years earlier isocyanates were used to graft polymers onto the surface of starch nanocrystals [105,106]. Since then, the use of isocyanates for nanocellulose modification has become more common. Unlike cellulose, nanocellulose modification took place only under heterogeneous conditions, which means that it mainly happened on the nanocellulose surface. Nanocellulose has up to 15% of its hydroxyls on the surface (excluding the unreactive C3 hydroxyls) [107,108,109]. This high percentage of surface hydroxyls makes nanocellulose more promising than cellulose for making functional cellulosic materials as it allows a more significant grafting of functional molecules on its surface. 

The reports on nanocellulose modification fell in the same four categories mentioned earlier for cellulose but with more focus on nanocellulose processing with thermoplastic and thermoset matrices (Table 2):

### 4.1. Preparation of Functional Nanocellulose (Scenario 1 and 3)

There are only three reports on nanocellulose functionalization. Similar to previous reports using cellulose, CNCs were reacted with 3,5-dimethylphenyl isocyanate to function as a stationary phase in liquid chromatography with chiral recognition abilities [29]. CNCs were also reacted with OI to function as a water-in-oil Pickering emulsifier [30]. Both reports followed Scenario 1 in Figure 4. Following Scenario 3, a photocleavable polymer was grafted on the surface of CNCs using 2,4-TDI as a linker [23].

### 4.2. Improving Nanocellulose Properties (Scenario 1,2, and 3)

Almost all the reports here focused on nanocellulose hydrophobization. CNFs were hydrophobized following Scenario 1 by reacting with OI [110] while CNCs were hydrophobized by reacting with castor oil and 2,4-TDI following Scenario 3 [45] and by reacting with poly(3-hydroxybutyrate-co-3-hydroxyvalerate) (PHBV) and 2,4-TDI following Scenario 4. The modified CNCs showed improved thermal stability and hydrophobicity [111]. CNFs with amine groups on the surface were prepared by reacting the CNFs with HMDI and certain alkyl diamines (Scenario 2) [112]. To improve its mechanical properties, a CNF aerogel was cross-linked with HMDI by immersing it in a solution of HMDI in acetone [113].

### 4.3. Improving Nanocellulose Processing and Performance with Nonpolar Matrices (Scenario 1, 2, and 3)

To be processed with PCL as a matrix, nanocellulose was modified using different approaches. In one approach, nanocellulose was reacted with OI following Scenario 1 and then mixed with PCL [39,40,114,115]. In the other approaches, PCL was grafted on the nanocellulose surface using 2,4-TDI following either Scenario 2 or 3 and the modified nanocellulose was then mixed with PCL as a matrix [43,104]. Nanocellulose was also processed with poly(butylene adipate-co-terephthalate) (PBAT) upon modification with OI or 4-phenylbutyl isocyanate (Scenario 1) [36,37,116,117]. Following Scenario 1 as well, nanocellulose was processed with PLA upon modification with OI or 2,4-TDI [42,118]. All these reports have shown an improved mechanical or thermomechanical composite performance upon nanocellulose modification due to an improved interfacial adhesion between the nanocellulose and the matrix. In terms of moisture/gas barrier properties and biodegradability, the results varied significantly.

### 4.4. Nanocellulose/Matrix Cross-Linking (Scenario 4)

The reports in this category cover the use of nanocellulose for reinforcing thermosets of different forms (foams, films, coatings), which were made of lignin, castor oil, poly(ethylene glycol) (PEG), polyether polyols, or other polyols. In some of these reports, the nanocellulose was added to the polyol/di-isocyanate mixture to crosslink all together [48,49,119]. In other reports, the nanocellulose was reacted with the di-isocyanate using one of its isocyanates then cross-linked with a mixture of the polyol with the same di-isocyanate [120,121,122,123,124,125,126,127]. An improvement in the mechanical or thermomechanical properties of the thermoset upon nanocellulose modification was the main outcome of these reports. An increase in the glass transition temperature of the thermoset was also observed.

## 5. Challenges

It is clear that mono- and di-isocyanates have great potential for nanocellulose modification. However, in addition to their reported toxicity, many challenges exist limiting their use [128,129]. One of the main challenges is that isocyanate reactions must take place in moisture-free environments. Otherwise, the isocyanates will get hydrolyzed and cross-linked to form a polyurea. This also requires the nanocellulose to be solvent exchanged to organic solvents prior to the reaction. This is in general problematic because nanocellulose, especially CNFs, tends to aggregate in these solvents affecting the homogeneity of the reaction. To minimize aggregation, ultrasonication is usually used, which is also problematic as it may, to a certain extent, degrade the nanocellulose and/or alter its surface properties [130]. 

Another issue is the difficulty of controlling the reaction between di-isocyanates and nanocellulose. Di-isocyanates are supposed to react with the surface hydroxyl groups of nanocellulose only through one of their two isocyanates (see Scenario 2 in Figure 4). In reality, this could be impossible to achieve as a significant fraction of the di-isocyanate reacts using both of its isocyanates becoming dysfunctional for any following grafting. This issue could be more challenging to resolve for HMDI compared to 2,4-TDI as the isocyanates of 2,4-TDI are not equally reactive. Moreover, the molecular flexibility of HMDI compared to 2,4-TDI increases the possibility of both of its isocyanates to react with nanocellulose. In terms of reactivity, the ortho isocyanate of 2,4-TDI is 5-10 times less reactive than the para one due to steric hindrance [33,34]. Despite this difference in reactivity, the reaction parameters have a major impact on para/ortho selectivity. Recently, Abushammala has developed a simple method to quantify the ortho isocyanates on the nanocellulose surface upon its reaction with 2,4-TDI [46]. Using this method, it was possible to optimize the reaction between 2,4-TDI and nanocellulose to obtain a maximum para/ortho selectivity of 93%, which means that 93% of the 2,4-TDI molecules reacted with the nanocellulose surface have their ortho isocyanates available for a following grafting [47]. The study also showed that the reaction temperature had a negative impact on selectivity as it minimizes the difference in the reaction kinetics of para and ortho isocyanates [131]. Another possibility to overcome this challenge is to follow Scenario 3 in Figure 4, which suggests the reaction of the di-isocyanate with the functional material or polymer at first then with the nanocellulose through the free isocyanate. Following this scenario, only the di-isocyanates, which have a free isocyanate, will be able to react with the nanocellulose. Those with two reacted isocyanates will be unreactive and therefore washed away after the reaction. 

The final challenge is the self-polymerization of isocyanates whether they are on the nanocellulose surface or in the reaction mixture. Isocyanates, in the presence of a catalyst and heat, can dimerize to uretidinediones and carbodiimides, or trimerize to isocyanurates, or even form larger oligomers. These many possibilities of isocyanates to self-polymerize have a negative impact on the efficiency of nanocellulose modification [132,133,134]. 

## 6. Conclusions

A variety of alkyl and aryl mono- and di-isocyanates have been used for the surface modification of nanocellulose. So far, many of these modifications focused on the compatibilization of nanocellulose with nonpolar thermoplastic and thermoset matrices for the fabrication of composites with enhanced interfacial adhesion. The main aim was improving the mechanical properties of the composites. Sometimes the impact of nanocellulose modification on the thermal properties of the composite, such as thermal stability and crystallization kinetics, was investigated. A few other modifications introduced functional groups on the nanocellulose surface to increase its potential in certain applications such as water filtration and biotechnological applications, etc. 

The reports on nanocellulose modification took place following one of four scenarios. In the first scenario, the nanocellulose was reacted in one-step with a mono-isocyanate (phenyl isocyanate or octadecyl isocyanate) to reduce the hydrophilicity of the nanocellulose. Sometimes, a di-isocyanate was used for the same purpose. In the second scenario, the nanocellulose was reacted with one of the isocyanates of a di-isocyanate then the free isocyanate was reacted with a functional material or a matrix. This scenario suffers the possibility of the di-isocyanate to react with the nanocellulose using both of its isocyanates becoming dysfunctional for the following step. The third scenario overcame this issue by reacting the di-isocyanate at first with the functional material or matrix using one of its isocyanates then the free isocyanate was reacted with the nanocellulose. The fourth scenario represents mainly the use of nanocellulose to reinforce nonpolar thermosets. In this scenario, the di-isocyanate was reacted with both the nanocellulose and the polyol at the same time.

Despite the reported potential of isocyanate compounds as chemical linkers and surface modifiers, their reactions with nanocellulose face many challenges including the need for moisture-free environment, nanocellulose aggregation during solvent-exchange, reaction controllability, and self-polymerization of isocyanates.

## Figures and Tables

**Table 1:** Summary of the reports on cellulose modification using mono- and di-isocyanates.

Category	Cellulose	Isocyanate	Matrix/Chemical	Ref
Functional Cellulose	Whatman Powder	2,4-TDI	-	[58]
	Cellulose Beads	2,4-TDI or HMDI	-	[59]
	MCC	2,4-TDI	n-Butanol	[44]
	Whatman Paper	Cyclohexyl Isocyanate or PI	-	[60]
	Cellulose	Phosphonium-containing Isocyanate	-	[61]
	Cotton Cellulose	Sulfopropylbetaine or Quaternary Ammonium Salt with a Reactive Isocyanate	-	[38]
	MCC	2,4-TDI	Amino acids	[62,63]
	Plant Cellulose	MDI	-	[64]
	Cotton Spheres	HMDI, 2,4-TDI, 1,4-PDI	-	[26]
	MCC	Substituted PI	-	[27]
	Cellulose Oligomers	3,5-Dimethylphenyl Isocyanate	-	[28]
	Cellulose	3-Chlorophenyl or 4-Chlorophenyl Isocyanate	Silica	[65]
Improving Cellulose Properties	Cellulose	PI, Cyclohexyl Isocyanate, or HMDI	-	[25]
	Cellulose	Oxime-blocked Isocyanate Oligomers	-	[66]
	Sisal Fibers	MDI	Soy-based Resin	[67]
	Six Celluloses	Alkenyl Isocyanates	Styrene or Methylacrylate	[68,69]
	Four Celluloses	Isocyanate-containing Polystyrene	-	[70,71]
	Cotton Fabric	2,4-TDI and PEG	-	[72]
	Cellophane Sheets	HMDI	Betaines	[73]
	Cellulose Fabric	2-Methacryloyloxyethyl Isocyanate	2-Methacryloyloxyethyl Phosphoryl Choline	[74]
	Cellulose Acetate	Phenyl, Propyl, or Butyl Isocyanate	-	[75,76]
Cellulose Processing with Nonpolar Matrices	Aspen Pulp and Sawdust	Poly(methylene(polyphenyl isocyanate))	PS	[77,78]
	Pine Pulp	MDI	PS	[79]
	Different Celluloses	Alkyl Isocyanates	PP	[80]
	Sisal Fibers	2,4-TDI	PP	[81]
	Whatman Fibers	HMDI	PP	[82]
	Bagasse Fibers	Polybutadiene Isocyanate	PP	[41]
	Birch Pulp	2,4-TDI	PP/PLA	[83]
	Pineapple Leaf Fibers	Poly(methylene(polyphenyl Isocyanate)) or HMDI	PP and PE	[84]
	MCC and Fibers	OI	PE	[69]
	Pineapple Leaf Cellulose	Poly(methylene(polyphenyl isocyanate))	PE	[85,86]
	Sisal Fibers	2,4-TDI-g-Cardanol	PE	[87,88]
	Pine Pulp	Derivatives of MDI	PP	[89]
	MCC	MDI and PPDI	Natural Rubber	[90]
	MCC	OI	Epoxidized Soybean Oil Polymer	[91]
	Hemp Fibers	3-Isopropenyl-dimethylbenzyl Isocyanate	Polyester	[92]
	Arbocell Fibers	MDI then Ethanol	Thermoplastic Urethane or a Polyamide	[93]
	Kenaf Fibers	Blocked MDI	Polyglycol Polyol, 1,4-Butanediol, and MDI	[94]
	Pulp Fibers	HMDI	Castor Oil	[95]
	Cellulose Acetate	HMDI	Castor Oil	[96]
	Eucalyptus Pulp	OI	Cement	[97,98]
Cellulose/ Matrix Cross-Linking	MCC and Pulp	2,4-TDI	PCL	[99]
	Cellulose Diacetate	2,4-TDI	Poly(caprolactone monoacrylate)	[100]
	Cellulose Diacetate	2,4-TDI	Poly(butylene glycol adipate)	[101]
	MCC	MDI	Castor Oil/MDI	[102]
	Cotton Cloth	Blocked Isocyanate of 2,4-TDI and Phenol	Lignin	[103]

**Table 2:** Summary of the reports on nanocellulose modification using mono- and di-isocyanates.

Category	CNCs/CNFs	Isocyanate	Matrix/Chemical	Ref
Functional Nanocellulose	CNCs	3,5-Dimethylphenyl Isocyanate	-	[29]
	CNCs	OI	-	[30]
	CNCs	2,4-TDI	Photocleavable Polymer	[23]
Improving Nanocellulose Properties	CNFs	OI	-	[110]
	CNCs	2,4-TDI	Castor Oil	[45]
	CNCs	2,4-TDI	PHBV	[111]
	CNFs	HMDI	Alkyl Diamines	[112]
	CNFs	HMDI	-	[113]
Nanocellulose Processing with Nonpolar Matrices	CNCs, CNFs	OI	PCL	[39,40]
	CNCs	OI	PCL	[114]
	CNCs	OI	PCL	[115]
	CNCs	2,4-TDI then PCL diol	PCL	[43]
	CNCs	PCL with 2,4-TDI	PCL	[104]
	CNCs	OI or 4-Phenylbutyl Isocyanate	PBAT	[36,37]
	CNCs	OI	PBAT	[116]
	CNCs	OI	PBAT	[117]
	CNCs	2,4-TDI	PLA	[42]
	CNCs	OI	PLA	[118]
Nanocellulose Matrix Cross-Linking	CNCs	MDI	Certain Polyols	[48,49]
	CNCs	HMDI	Polyurethane	[119]
	CNFs	Polymeric MDI	Lignin-Soy Polyol with Polymer MDI	[120]
	CNCs	MDI	Castor Oil and MDI	[121]
	CNCs	Isophorone Diisocyanate	Isophorone Diisocyanate and a Trifunctional Polyether Alcohol	[122]
	CNFs	Methylenebis(Cyclohexyl Isocyanate)	Methylenebis(Cyclohexyl Isocyanate) with PEG	[123]
	CNFs	MDI	Castor Oil Polyol and MDI	[124]
	CNFs	Poly(phenyl Isocyanate)	PEG and Poly(methylene(polyphenyl isocyanate))	[125]
	CNCs	Polymeric MDI	Polyether Polyol and Polymeric MDI	[126]
	CNCs	Photocurable Isocyanate (3-isopropenyl-α,α-dimethylbenzyl Isocyanate)	Polyether Polyol and 3-Isopropenyl-α,α-dimethylbenzyl Isocyanate	[127]

## References

[1] Sun R. (2010). Cereal Straw as a Resource for Sustainable Biomaterials and Biofuels: Chemistry, Extractives, Lignins, Hemicelluloses and Cellulose.

[2] Spence K.L., Venditti R.A., Rojas O.J., Habibi Y., Pawlak J.J. (2010). The effect of chemical composition on microfibrillar cellulose films from wood pulps: Water interactions and physical properties for packaging applications. Cellulose.

[3] Mao J., Abushammala H., Brown N., Laborie M.-P. (2017). Comparative assessment of methods for producing cellulose I nanocrystals from cellulosic sources. Nanocelluloses: Their Preparation, Properties, and Applications, ACS Symposium Series.

[4] (2017). Standard Terms and Their Definition for Cellulose Nanomaterial.

[5] Bondeson D., Mathew A., Oksman K. (2006). Optimization of the isolation of nanocrystals from microcrystalline cellulose by acid hydrolysis. Cellulose.

[6] Abushammala H., Goldsztayn R., Leao A., Laborie M.-P. (2016). Combining steam explosion with 1-ethyl-3-methylimidazlium acetate treatment of wood yields lignin-coated cellulose nanocrystals of high aspect ratio. Cellulose.

[7] Abushammala H., Krossing I., Laborie M.-P. (2015). Ionic liquid-mediated technology to produce cellulose nanocrystals directly from wood. Carbohydr. Polym..

[8] Leung C.W., Luong J.H., Hrapovic S., Lam E., Liu Y., Male K.B., Mahmoud K., Rho D. (2014). Cellulose nanocrystals from renewable biomass. U.S. Patent.

[9] Sacui I.A., Nieuwendaal R.C., Burnett D.J., Stranick S.J., Jorfi M., Weder C., Foster E.J., Olsson R.T., Gilman J.W. (2014). Comparison of the properties of cellulose nanocrystals and cellulose nanofibrils isolated from bacteria, tunicate, and wood processed using acid, enzymatic, mechanical, and oxidative methods. Acs Appl. Mater. Interfaces.

[10] Moon R.J., Martini A., Nairn J., Simonsen J., Youngblood J. (2011). Cellulose nanomaterials review: Structure, properties and nanocomposites. Chem. Soc. Rev..

[11] Kiziltas A., Erbas Kiziltas E., Boran S., Gardner D.J. Micro-and nanocellulose composites for automotive applications. Proceedings of the SPE Automotive Composites Conference and Exhibition (ACCE).

[12] Plackett D., Letchford K., Jackson J., Burt H. (2014). A review of nanocellulose as a novel vehicle for drug delivery. Nord. Pulp. Pap. Res. J..

[13] Dugan J.M., Gough J.E., Eichhorn S.J. (2013). Bacterial cellulose scaffolds and cellulose nanowhiskers for tissue engineering. Nanomed..

[14] Khan A., Huq T., Khan R.A., Riedl B., Lacroix M. (2014). Nanocellulose-based composites and bioactive agents for food packaging. Crit. Rev. Food Sci. Nutr..

[15] Voisin H., Bergström L., Liu P., Mathew A. (2017). Nanocellulose-based materials for water purification. Nanomaterials.

[16] Fraschini C., Chauve G., Bouchard J. (2017). TEMPO-mediated surface oxidation of cellulose nanocrystals (CNCs). Cellulose.

[17] Wu Z., Xu J., Gong J., Li J., Mo L. (2018). Preparation, characterization and acetylation of cellulose nanocrystal allomorphs. Cellulose.

[18] Yuan H., Nishiyama Y., Wada M., Kuga S. (2006). Surface acylation of cellulose whiskers by drying aqueous emulsion. Biomacromolecules.

[19] Salajková M., Berglund L.A., Zhou Q. (2012). Hydrophobic cellulose nanocrystals modified with quaternary ammonium salts. J. Mater. Chem..

[20] Song Z., Xiao H., Zhao Y. (2014). Hydrophobic-modified nano-cellulose fiber/PLA biodegradable composites for lowering water vapor transmission rate (WVTR) of paper. Carbohydr. Polym..

[21] Cervin N.T., Aulin C., Larsson P.T., Wågberg L. (2012). Ultra porous nanocellulose aerogels as separation medium for mixtures of oil/water liquids. Cellulose.

[22] Laitinen O., Hartmann R., Sirviö J.A., Liimatainen H., Rudolph M., Ämmälä A., Illikainen M. (2016). Alkyl aminated nanocelluloses in selective flotation of aluminium oxide and quartz. Chem. Eng. Sci..

[23] Morandi G., Thielemans W. (2012). Synthesis of cellulose nanocrystals bearing photocleavable grafts by ATRP. Polym. Chem..

[24] Akindoyo J.O., Beg M., Ghazali S., Islam M., Jeyaratnam N., Yuvaraj A. (2016). Polyurethane types, synthesis and applications–a review. RSC Adv..

[25] Zhang C., Gilbert R., Fornes R. (1992). Preliminary studies of reduction of moisture absorption of cellulose using masked isocyanates. Abstracts of Papers of the American Chemical Society.

[26] Chen W., Bin Q., Bai Z.-W., Zhou X.-P., Xie X.-L. (2013). Partial carbamoylation of cellulose microspheres: A new method to prepare adsorbents for liquid chromatography. Chin. J. Polym. Sci..

[27] Chen W., Zhang M., Feng Y., Wu J., Gao X., Zhang J., He J., Zhang J. (2015). Homogeneous synthesis of partially substituted cellulose phenylcarbamates aiming at chiral recognition. Polym. Int..

[28] Okada Y., Yamamoto C., Kamigaito M., Gao Y., Shen J., Okamoto Y. (2016). Enantioseparation using cellulose tris (3, 5-dimethylphenylcarbamate) as chiral stationary phase for HPLC: Influence of molecular weight of cellulose. Molecules.

[29] Zhang X., Wang L., Dong S., Zhang X., Wu Q., Zhao L., Shi Y. (2016). Nanocellulose 3, 5-Dimethylphenylcarbamate Derivative Coated Chiral Stationary Phase: Preparation and Enantioseparation Performance. Chirality.

[30] Guo J., Du W., Gao Y., Cao Y., Yin Y. (2017). Cellulose nanocrystals as water-in-oil Pickering emulsifiers via intercalative modification. Colloids Surf. A Physicochem. Eng. Asp..

[31] Guo Y.-H., Guo J.-J., Li S.-C., Li X., Wang G.-S., Huang Z. (2013). Properties and paper sizing application of waterborne polyurethane emulsions synthesized with TDI and IPDI. Colloids Surf. A Physicochem. Eng. Asp..

[32] Mix R., Gähde J., Goering H., Schulz G. (1996). Segmented polyurethanes with 4, 4′-bis-(6-hydroxyhexoxy) biphenyl as chain extender. Part 2. Synthesis and properties of MDI-polyurethanes in comparison with 2, 4-TDI-polyurethanes. J. Polym. Sci. Part A Polym. Chem..

[33] Belgacem M.N., Quillerou J., Gandini A. (1993). Urethanes and polyurethanes bearing furan moieties—3. Synthesis, characterization and comparative kinetics of the formation of diurethanes. Eur. Polym. J..

[34] Semsarzadeh M., Navarchian A. (2003). Kinetic Study of the Bulk Reaction Between TDI and PPG in Prescence of DBTDL and FEAA Catalysts Using Quantitative FTIR Spectroscopy. J. Polym. Eng..

[35] Evans R., Wearne R.H., Wallis A.F. (1991). Effect of amines on the carbanilation of cellulose with phenylisocyanate. J. Appl. Polym. Sci..

[36] Morelli C.L., Belgacem N., Bretas R.E., Bras J. (2016). Melt extruded nanocomposites of polybutylene adipate-co-terephthalate (PBAT) with phenylbutyl isocyanate modified cellulose nanocrystals. J. Appl. Polym. Sci..

[37] Morelli C.L., Belgacem M.N., Branciforti M.C., Bretas R.E., Crisci A., Bras J. (2016). Supramolecular aromatic interactions to enhance biodegradable film properties through incorporation of functionalized cellulose nanocrystals. Compos. Part A Appl. Sci. Manuf..

[38] Zhang S., Yang X., Tang B., Yuan L., Wang K., Liu X., Zhu X., Li J., Ge Z., Chen S. (2018). New insights into synergistic antimicrobial and antifouling cotton fabrics via dually finished with quaternary ammonium salt and zwitterionic sulfobetaine. Chem. Eng. J..

[39] Siqueira G., Bras J., Dufresne A. (2009). New process of chemical grafting of cellulose nanoparticles with a long chain isocyanate. Langmuir.

[40] Siqueira G., Bras J., Follain N., Belbekhouche S., Marais S., Dufresne A. (2013). Thermal and mechanical properties of bio-nanocomposites reinforced by Luffa cylindrica cellulose nanocrystals. Carbohydr. Polym..

[41] Ashori A., Nourbakhsh A. (2009). Polypropylene cellulose-based composites: The effect of bagasse reinforcement and polybutadiene isocyanate treatment on the mechanical properties. J. Appl. Polym. Sci..

[42] Gwon J.-G., Cho H.-J., Chun S.-J., Lee S., Wu Q., Lee S.-Y. (2016). Physiochemical, optical and mechanical properties of poly (lactic acid) nanocomposites filled with toluene diisocyanate grafted cellulose nanocrystals. RSC Adv..

[43] Zoppe J.O., Peresin M.S., Habibi Y., Venditti R.A., Rojas O.J. (2009). Reinforcing poly (ε-caprolactone) nanofibers with cellulose nanocrystals. ACS Appl. Mater. Interfaces.

[44] Dierov Z.K., Tsiomenko A., Davranov K., Kulaev I. (1993). Hydrophobic chromatography and characterization of lipases secreted by the fungus rhizopus-microporous UZLT-4B. Biochem. -Mosc..

[45] Shang W., Huang J., Luo H., Chang P.R., Feng J., Xie G. (2013). Hydrophobic modification of cellulose nanocrystal via covalently grafting of castor oil. Cellulose.

[46] Abushammala H. (2019). A Simple Method for the Quantification of Free Isocyanates on the Surface of Cellulose Nanocrystals upon Carbamation using Toluene Diisocyanate. Surface.

[47] Abushammala H. (2019). On the Para/Ortho Reactivity of Isocyanate Groups during the Carbamation of Cellulose Nanocrystals Using 2,4-Toluene Diisocyanate. Polymer.

[48] Li Y., Ren H., Ragauskas A.J. (2011). Rigid polyurethane foam/cellulose whisker nanocomposites: Preparation, characterization, and properties. J. Nanosci. Nanotechnol..

[49] Li Y., Ragauskas A.J. (2012). Ethanol organosolv lignin-based rigid polyurethane foam reinforced with cellulose nanowhiskers. RSC Adv..

[50] Charles G.P.E. (1920). Manufacture of new products derived from cellulose. U.S. Patent.

[51] Welch C.M. (1961). Process for the reaction of isocyanates with cellulose in the presence of organic phosphites. U.S. Patent.

[52] George M. (1947). Structural element made from paper and like sheets. U.S. Patent.

[53] Ellzey S., Wade C.P., Mack C.H. (1962). Part II: Textile Properties of Fabric Modified by Reaction with Phenyl Isocyanate. Text. Res. J..

[54] Ellzey S., Mack C.H. (1962). Reaction of Aryl Isocyanates with Cotton Cellulose: Part I: Variables in the Reaction Using Phenyl Isocyanate. Text. Res. J..

[55] Ohno Y., Uchimoto I. (1970). Studies on reaction of cellulose with isocyanate. 1. Reaction of cellulose with phenyl isocyanate. Kog Kagaku Zasshi.

[56] Ohno Y., Sato T., Miyamoto K. (1976). Studies on reaction of cellulose with isocyanate. 3. Reaction of cellulose with 2, 4-diisocyanatotoluene in n, n-dimethylformamide. Nippon. Kagaku. Kaishi.

[57] Sato T., Ohno Y., Tamura T. (1978). Studies on reaction of cellulose with isocyanate. 5. Reaction of cellulose with hexamethylene diisocyanate in n, n-dimethylformamide. Nippon. Kagaku. Kaishi.

[58] Gemeiner P., Augustin J., Drobnica L. (1977). Reactions of cellulose isothiocyanates with thiol and amino compounds. Carbohydr. Res..

[59] Chen L.F., Tsao G.T. (1977). Chemical procedures for enzyme immobilization on porous cellulose beads. Biotechnol. Bioeng..

[60] Saraji M., Farajmand B. (2013). Chemically modified cellulose paper as a thin film microextraction phase. J. Chromatogr. A.

[61] Pend X., Sato M., Kawase T., Ikeno K., Sawada H., Hamada N., Wada K., Takahashi Y., Yoshimura T. (2002). Synthesis and soil repellent, antibacterial and antifungal properties of blocked isocyanate co-oligomers having cation segments. Sen-I Gakkaishi.

[62] Sato T., Karatsu K., Kitamura H., Ohno Y. (1983). Synthesis of cellulose derivatives containing amino acid residues and their adsorption of metal ions. Sen’i Gakkaishi.

[63] Sato T., Motomura S., Ohno Y. (1985). Adsorption and desorption of metal ions by systems based on cellulose derivatives that contain amino acid residues. Sen’i Gakkaishi.

[64] Tursi A., Beneduci A., Chidichimo F., De Vietro N., Chidichimo G. (2018). Remediation of hydrocarbons polluted water by hydrophobic functionalized cellulose. Chemosphere.

[65] Sato J., Sugimura K., Teramoto Y., Nishio Y. (2019). Preparation and chiroptical properties of cellulose chlorophenylcarbamate–silica hybrids having a chiral nematic mesomorphic structure. Polymer.

[66] Peng X., Kawase T., Sato M., Ikeno K., Sawada H. (2002). Surface modification of cellulose and polyester by oligomeric fluoroalkylating agents having oxime-blocked isocyanate groups. Sen-I Gakkaishi.

[67] Rajkumar S., Tjong J., Nayak S., Sain M. (2015). Wetting behavior of soy-based resin and unsaturated polyester on surface-modified sisal fiber mat. J. Reinf. Plast. Compos..

[68] Botaro V.R., Gandini A. (1998). Chemical modification of the surface of cellulosic fibres. 2. Introduction of alkenyl moieties via condensation reactions involving isocyanate functions. Cellulose.

[69] Botaro V.R., Gandini A., Belgacem M.N. (2005). Heterogeneous chemical modification of cellulose for composite materials. J. Thermoplast. Compos. Mater..

[70] Trejo-O’Reilly J., Cavaille J.Y., Gandini A. (1997). Cationic copolymerization of styrenes with an isocyanate-bearing homologue. React. Funct. Polym..

[71] Trejo-O’reilly J.-A., Cavaille J.-Y., Gandini A. (1997). The surface chemical modification of cellulosic fibres in view of their use in composite materials. Cellulose.

[72] Badanova A.K., Taussarova B.R., Kutzhanova A.Z. (2014). Hydrophobic finishing of cellulosic textile material. World. Appl. Sci. J..

[73] Yuan J., Zhang J., Zang X., Shen J., Lin S. (2003). Improvement of blood compatibility on cellulose membrane surface by grafting betaines. Colloids. Surf. B Biointerfaces.

[74] Furuzono T., Ishihara K., Nakabayashi N., Tamada Y. (2000). Chemical modification of silk fibroin with 2-methacryloyloxyethyl phosphorylcholine. II. Graft-polymerization onto fabric through 2-methacryloyloxyethyl isocyanate and interaction between fabric and platelets. Biomaterials.

[75] Ghatge N., Sabne M., Gujar K., Mahajan S. (1984). Modification of cellulose acetate by aliphatic isocyanates for reverse osmosis studies. Int. J. Polym. Mater..

[76] Mahajan S., Sabne M., Gujar K., Ghatge N. (1985). Selectivity of isocyanate modified cellulose acetate membranes to sugars. Int. J. Polym. Mater..

[77] Maldas D., Kokta B.V. (1990). Effect of Fiber Treatment on the Mechanical Properties of Hybrid Fiber-Reinforced Polystyrene Composites: I. Use of Mica and Wood Pulp as Hybrid Filler. J. Compos. Technol. Res..

[78] Maldas D., Kokta B. (1991). Effect of fiber treatment on the mechanical properties of hybrid fiber reinforced polystyrene composites: IV. Use of glass fiber and sawdust as hybrid fiber. J. Compos. Mater..

[79] Girones J., Pimenta M., Vilaseca F., De Carvalho A., Mutje P., Curvelo A. (2007). Blocked isocyanates as coupling agents for cellulose-based composites. Carbohydr. Polym..

[80] Joly C., Kofman M., Gauthier R. (1996). Polypropylene/cellulosic fiber composites chemical treatment of the cellulose assuming compatibilization between the two materials. J. Macromol. Sci. Part A Pure Appl. Chem..

[81] Canche-Escamilla G., Cauich-Cupul J., Mendizabal E., Puig J., Vazquez-Torres H., Herrera-Franco P. (1999). Mechanical properties of acrylate-grafted henequen cellulose fibers and their application in composites. Compos. Part A Appl. Sci. Manuf..

[82] Qiu W., Zhang F., Endo T., Hirotsu T. (2005). Isocyanate as a compatibilizing agent on the properties of highly crystalline cellulose/polypropylene composites. J. Mater. Sci..

[83] Darie R.N., Vlad S., Anghel N., Doroftei F., Tamminen T., Spiridon I. (2015). New PP/PLA/cellulose composites: Effect of cellulose functionalization on accelerated weathering behavior. Polym. Adv. Technol..

[84] Suwanruji P., Tuechart T., Smitthipong W., Chollakup R. (2017). Modification of pineapple leaf fiber surfaces with silane and isocyanate for reinforcing thermoplastic. J. Thermoplast. Compos. Mater..

[85] George J., Bhagawan S., Thomas S. (1997). Improved interactions in chemically modified pineapple leaf fiber reinforced polyethylene composites. Compos. Interfaces.

[86] George J., Bhagawan S., Thomas S. (1996). Thermogravimetric and dynamic mechanical thermal analysis of pineapple fibre reinforced polyethylene composites. J. Therm. Anal. Calorim..

[87] Joseph K., Thomas S., Pavithran C. (1996). Effect of chemical treatment on the tensile properties of short sisal fibre-reinforced polyethylene composites. Polymer.

[88] Joseph P., Rabello M.S., Mattoso L., Joseph K., Thomas S. (2002). Environmental effects on the degradation behaviour of sisal fibre reinforced polypropylene composites. Compos. Sci. Technol..

[89] Girones J., Pimenta M., Vilaseca F., Carvalho A.J.d., Mutje P., Curvelo A. (2008). Blocked diisocyanates as reactive coupling agents: Application to pine fiber–polypropylene composites. Carbohydr. Polym..

[90] Ly B., Thielemans W., Dufresne A., Chaussy D., Belgacem M. (2008). Surface functionalization of cellulose fibres and their incorporation in renewable polymeric matrices. Compos. Sci. Technol..

[91] Zhang S., Xia C., Dong Y., Yan Y., Li J., Shi S.Q., Cai L. (2016). Soy protein isolate-based films reinforced by surface modified cellulose nanocrystal. Ind. Crop. Prod..

[92] Liu W., Chen T., Qiu R. (2014). Effect of fiber modification with 3-isopropenyl-dimethylbenzyl isocyanate (TMI) on the mechanical properties and water absorption of hemp-unsaturated polyester (UPE) composites. Holzforschung.

[93] Reulier M., Perrin R., Avérous L. (2016). Biocomposites based on chemically modified cellulose fibers with renewable fatty-acid-based thermoplastic systems: Effect of different fiber treatments. J. Appl. Polym. Sci..

[94] Datta J., Kopczyńska P. (2015). Effect of kenaf fibre modification on morphology and mechanical properties of thermoplastic polyurethane materials. Ind. Crop. Prod..

[95] Gallego R., Arteaga J., Valencia C., Franco J. (2015). Thickening properties of several NCO-functionalized cellulose derivatives in castor oil. Chem. Eng. Sci..

[96] Tenorio-Alfonso A., Sánchez M.C., Franco J.M. (2017). Preparation, characterization and mechanical properties of bio-based polyurethane adhesives from isocyanate-functionalized cellulose acetate and castor oil for bonding wood. Polymer.

[97] Tonoli G.H.D., Mendes R.F., Siqueira G., Bras J., Belgacem M.N., Savastano H. (2013). Isocyanate-treated cellulose pulp and its effect on the alkali resistance and performance of fiber cement composites. Holzforschung.

[98] Tonoli G.H.D., Belgacem M.N., Siqueira G., Bras J., Savastano Jr H., Lahr F.R. (2013). Processing and dimensional changes of cement based composites reinforced with surface-treated cellulose fibres. Cem. Concr. Compos..

[99] Paquet O., Krouit M., Bras J., Thielemans W., Belgacem M.N. (2010). Surface modification of cellulose by PCL grafts. Acta Mater..

[100] Wang D., Xuan Y., Huang Y., Shen J. (2003). Synthesis and properties of graft copolymer of cellulose diacetate with poly (caprolactone monoacrylate). J. Appl. Polym. Sci..

[101] Xu L., Cheng X. (2014). Preparation and characterization of cellulose diacetate-graft-poly (butylene glycol adipate) copolymers. Russ. J. Appl. Chem..

[102] Miao S.D., Liu Y.Y., Wang P., Zhang S.P. (2012). Castor oil and microcrystalline cellulose based polymer composites with high tensile strength. Adv. Mater. Res..

[103] Cardamone J.M. (1992). Reacting cotton cellulose with lignin-based polyurethane. Text. Res. J..

[104] Habibi Y., Dufresne A. (2008). Highly filled bionanocomposites from functionalized polysaccharide nanocrystals. Biomacromolecules.

[105] Labet M., Thielemans W., Dufresne A. (2007). Polymer grafting onto starch nanocrystals. Biomacromolecules.

[106] Thielemans W., Belgacem M.N., Dufresne A. (2006). Starch nanocrystals with large chain surface modifications. Langmuir.

[107] Gu J., Catchmark J.M., Kaiser E.Q., Archibald D.D. (2013). Quantification of cellulose nanowhiskers sulfate esterification levels. Carbohydr. Polym..

[108] Nishiyama Y., Langan P., Chanzy H. (2002). Crystal structure and hydrogen-bonding system in cellulose Iβ from synchrotron X-ray and neutron fiber diffraction. J. Am. Chem. Soc..

[109] Verlhac C., Dedier J., Chanzy H. (1990). Availability of surface hydroxyl groups in Valonia and bacterial cellulose. J. Polym. Sci. Part A Polym. Chem..

[110] Missoum K., Bras J., Belgacem M.N. (2012). Organization of aliphatic chains grafted on nanofibrillated cellulose and influence on final properties. Cellulose.

[111] Yu H.-Y., Qin Z.-Y. (2014). Surface grafting of cellulose nanocrystals with poly (3-hydroxybutyrate-co-3-hydroxyvalerate). Carbohydr. Polym..

[112] Stenstad P., Andresen M., Tanem B.S., Stenius P. (2008). Chemical surface modifications of microfibrillated cellulose. Cellulose.

[113] Verdolotti L., Stanzione M., Khlebnikov O., Silant’ev V., Postnova I., Lavorgna M., Shchipunov Y. (2019). Dimensionally Stable Cellulose Aerogel Strengthened by Polyurethane Synthesized in Situ. Macromol. Chem. Phys..

[114] Hassan M.L., Bras J., Hassan E.A., Fadel S.M., Dufresne A. (2012). Polycaprolactone/modified bagasse whisker nanocomposites with improved moisture-barrier and biodegradability properties. J. Appl. Polym. Sci..

[115] Follain N., Belbekhouche S., Bras J., Siqueira G., Chappey C., Marais S., Dufresne A. (2018). Tunable gas barrier properties of filled-PCL film by forming percolating cellulose network. Colloids Surf. A Physicochem. Eng. Asp..

[116] Pinheiro I., Ferreira F., Souza D., Gouveia R., Lona L., Morales A., Mei L. (2017). Mechanical, rheological and degradation properties of PBAT nanocomposites reinforced by functionalized cellulose nanocrystals. Eur. Polym. J..

[117] Pinheiro I., Ferreira F., Alves G., Rodolfo A., Morales A., Mei L. (2019). Biodegradable PBAT-Based Nanocomposites Reinforced with Functionalized Cellulose Nanocrystals from Pseudobombax munguba: Rheological, Thermal, Mechanical and Biodegradability Properties. J. Polym. Environ..

[118] Espino-Pérez E., Bras J., Ducruet V., Guinault A., Dufresne A., Domenek S. (2013). Influence of chemical surface modification of cellulose nanowhiskers on thermal, mechanical, and barrier properties of poly (lactide) based bionanocomposites. Eur. Polym. J..

[119] Rueda L., d’Arlas B.F., Zhou Q., Berglund L.A., Corcuera M., Mondragon I., Eceiza A. (2011). Isocyanate-rich cellulose nanocrystals and their selective insertion in elastomeric polyurethane. Compos. Sci. Technol..

[120] Faruk O., Sain M., Farnood R., Pan Y., Xiao H. (2014). Development of lignin and nanocellulose enhanced bio PU foams for automotive parts. J. Polym. Environ..

[121] Cordero A.I., Amalvy J.I., Fortunati E., Kenny J.M., Chiacchiarelli L.M. (2015). The role of nanocrystalline cellulose on the microstructure of foamed castor-oil polyurethane nanocomposites. Carbohydr. Polym..

[122] Girouard N.M., Xu S., Schueneman G.T., Shofner M.L., Meredith J.C. (2016). Site-selective modification of cellulose nanocrystals with isophorone diisocyanate and formation of polyurethane-CNC composites. ACS Appl. Mater. Interfaces.

[123] Ikhwan F., Ilmiati S., Adi H.K., Arumsari R., Chalid M. Novel route of synthesis for cellulose fiber-based hybrid polyurethane. Proceedings of the Innovation in Polymer Science and Technology.

[124] Gimenez R.B., Leonardi L., Cerrutti P., Amalvy J., Chiacchiarelli L.M. (2017). Improved specific thermomechanical properties of polyurethane nanocomposite foams based on castor oil and bacterial nanocellulose. J. Appl. Polym. Sci..

[125] Leng W., Li J., Cai Z. (2017). Synthesis and characterization of cellulose nanofibril-reinforced polyurethane foam. Polymers.

[126] Kong X., Wolodko J., Zhao L., Curtis J.M. (2018). The preparation and characterization of polyurethane reinforced with a low fraction of cellulose nanocrystals. Prog. Org. Coat..

[127] Hubmann M., Kong X., Curtis J.M. (2019). Kinetic stabilization of cellulose nanocrystals in a photocurable prepolymer for application as an adhesion promoter in UV-curable coatings. Prog. Org. Coat..

[128] Musk A.W., Peters J.M., Wegman D.H. (1988). Isocyanates and respiratory disease: Current status. Am. J. Ind. Med..

[129] Bengtström L., Salden M., Stec A.A. (2016). The role of isocyanates in fire toxicity. Fire. Sci. Rev..

[130] Marx-Figini M. (1997). Studies on the ultrasonic degradation of cellulose macromolecular properties. Die Angew. Makromol. Chem. Appl. Macromol. Chem. Phys..

[131] Aranguren M.I., Williams R.J. (1986). Kinetic and statistical aspects of the formation of polyurethanes from toluene diisocyanate. Polymer.

[132] Buckles R.E., McGrew L. (1966). A kinetic study of the dimerization of phenyl isocyanate. J. Am. Chem. Soc..

[133] Schwetlick K., Noack R. (1995). Kinetics and catalysis of consecutive isocyanate reactions. Formation of carbamates, allophanates and isocyanurates. J. Chem. Soc. Perkin Trans. 2.

[134] Guo J., He Y., Xie D., Zhang X. (2015). Process investigating and modelling for the self-polymerization of toluene diisocyanate (TDI)-based polyurethane prepolymer. J. Mater. Sci..

